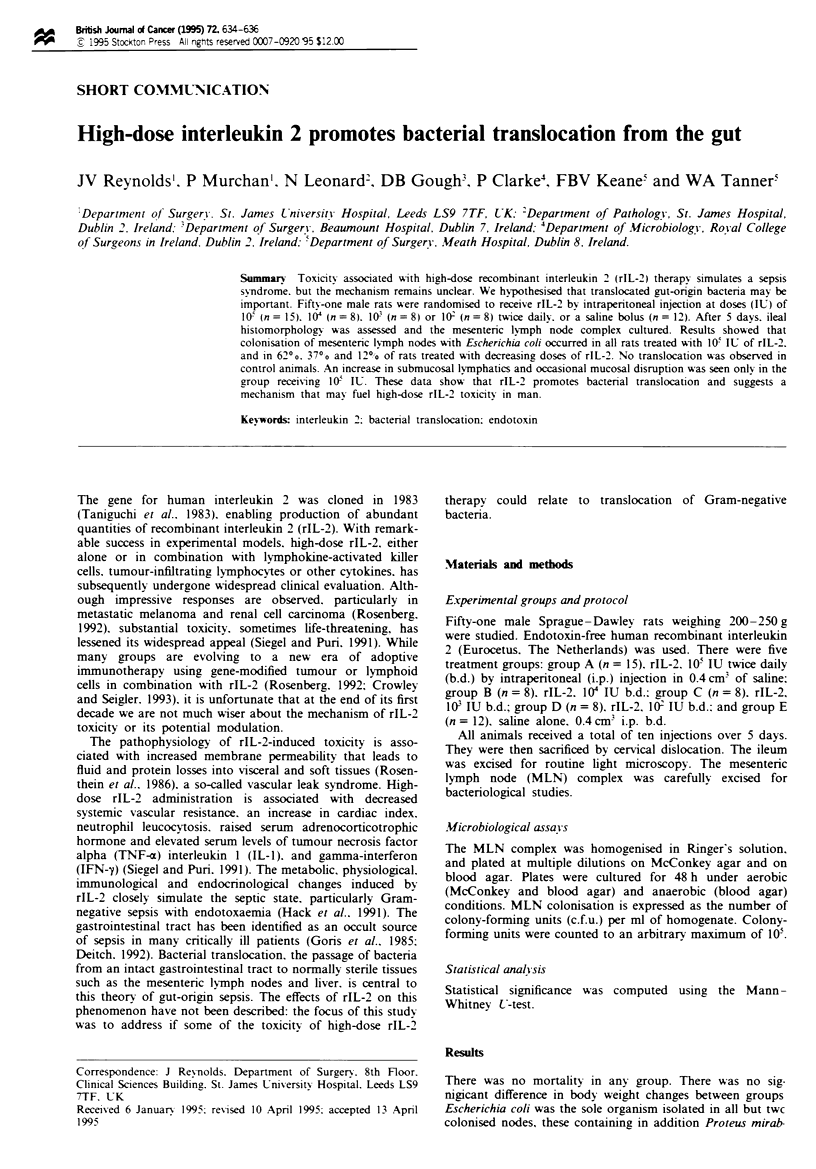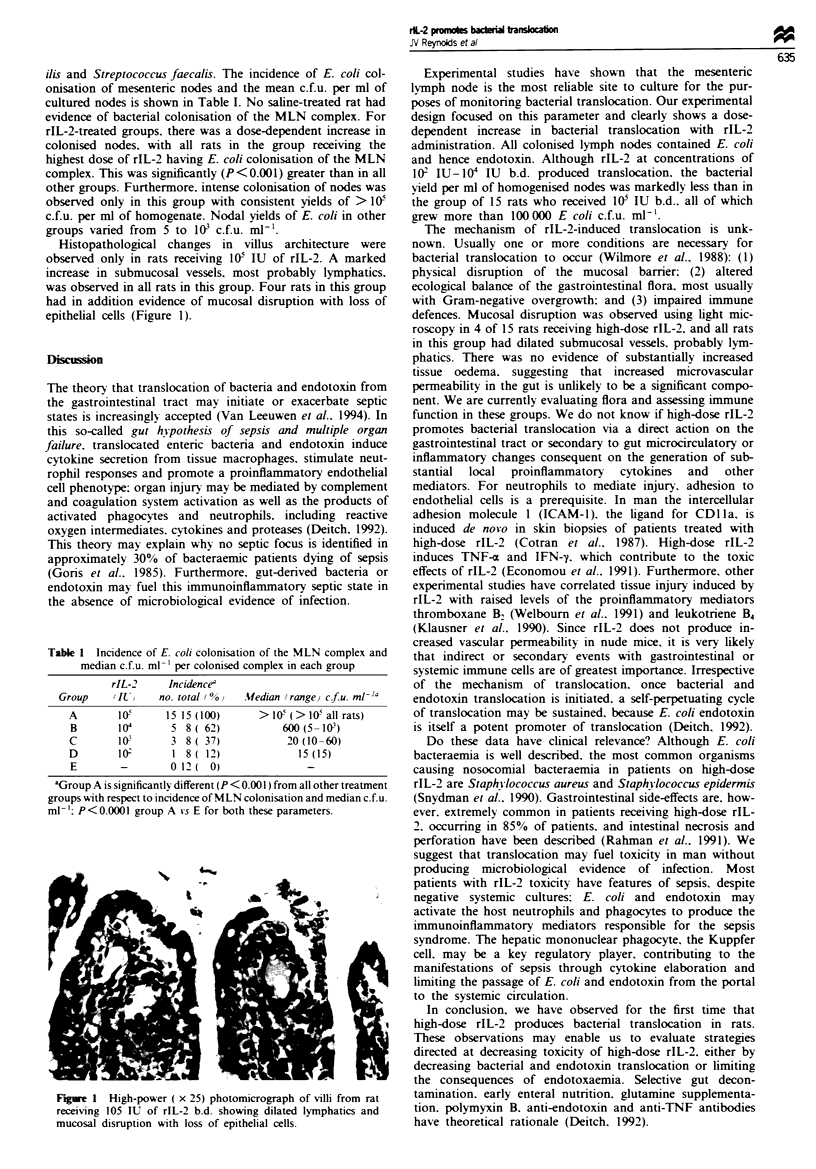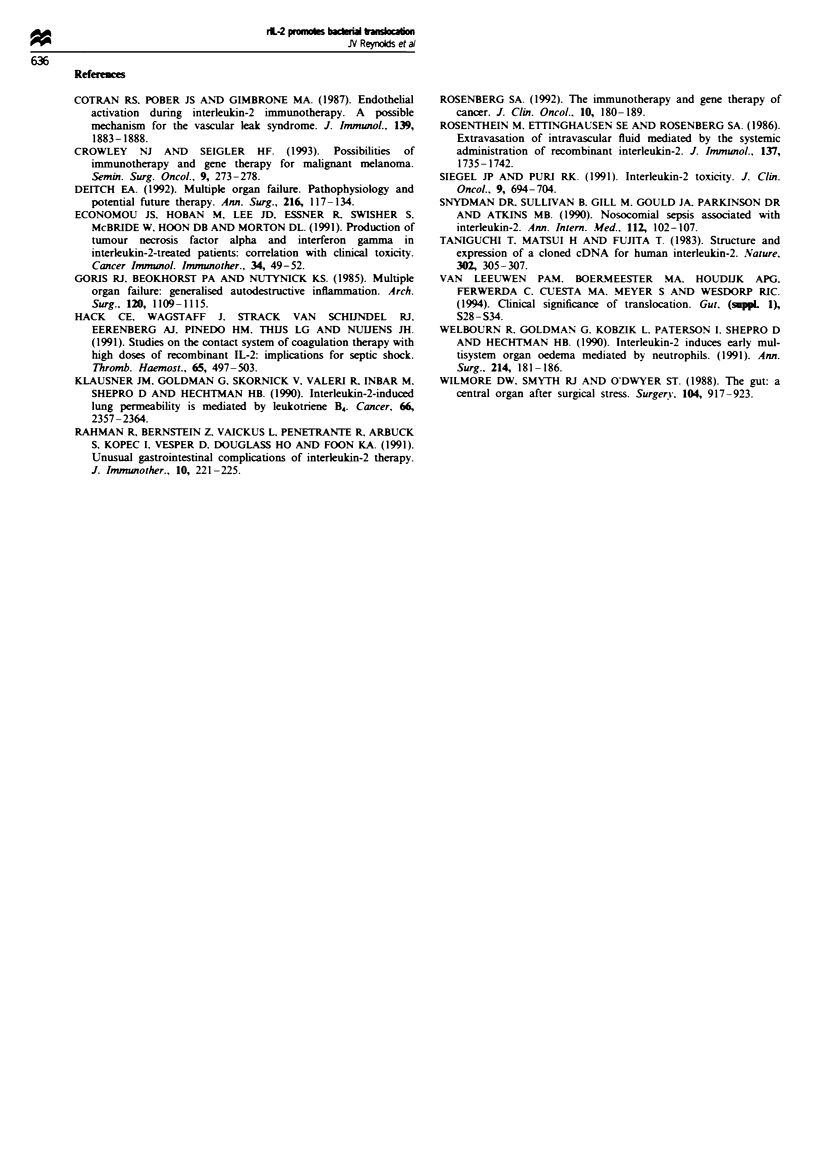# High-dose interleukin 2 promotes bacterial translocation from the gut.

**DOI:** 10.1038/bjc.1995.385

**Published:** 1995-09

**Authors:** J. V. Reynolds, P. Murchan, N. Leonard, D. B. Gough, P. Clarke, F. B. Keane, W. A. Tanner

**Affiliations:** Department of Surgery, St. James University Hospital, Leeds, UK.

## Abstract

**Images:**


British Journal of Cancer (1995) 72, 634–636

© 1995 Stockton Press All rights reserved 0007-0920/95 $12.00

SHORT COMMUNICATION

### High-dose interleukin 2 promotes bacterial translocation from the gut

JV Reynolds[1], P Murchan[1], N Leonard[2], DB Gough[3], P Clarke[4], FBV Keane[5] and WA Tanner[5]

[1]*Department of Surgery, St. James University Hospital, Leeds LS9 7TF, UK;* [2]*Department of Pathology, St. James Hospital, Dublin 2, Ireland;* [3]*Department of Surgery, Beaumount Hospital, Dublin 7, Ireland;* [4]*Department of Microbiology, Royal College of Surgeons in Ireland, Dublin 2, Ireland;* [5]*Department of Surgery, Meath Hospital, Dublin 8, Ireland.*

**Summary** Toxicity associated with high-dose recombinant interleukin 2 (rIL-2) therapy simulates a sepsis syndrome, but the mechanism remains unclear. We hypothesised that translocated gut-origin bacteria may be important. Fifty-one male rats were randomised to receive rIL-2 by intraperitoneal injection at doses (IU) of $10^5$ ($n = 15$), $10^4$ ($n = 8$), $10^3$ ($n = 8$) or $10^2$ ($n = 8$) twice daily, or a saline bolus ($n = 12$). After 5 days, ileal histomorphology was assessed and the mesenteric lymph node complex cultured. Results showed that colonisation of mesenteric lymph nodes with *Escherichia coli* occurred in all rats treated with $10^5$ IU of rIL-2, and in 62%, 37% and 12% of rats treated with decreasing doses of rIL-2. No translocation was observed in control animals. An increase in submucosal lymphatics and occasional mucosal disruption was seen only in the group receiving $10^5$ IU. These data show that rIL-2 promotes bacterial translocation and suggests a mechanism that may fuel high-dose rIL-2 toxicity in man.

**Keywords:** interleukin 2; bacterial translocation; endotoxin

The gene for human interleukin 2 was cloned in 1983 (Taniguchi *et al*., 1983), enabling production of abundant quantities of recombinant interleukin 2 (rIL-2). With remarkable success in experimental models, high-dose rIL-2, either alone or in combination with lymphokine-activated killer cells, tumour-infiltrating lymphocytes or other cytokines, has subsequently undergone widespread clinical evaluation. Although impressive responses are observed, particularly in metastatic melanoma and renal cell carcinoma (Rosenberg, 1992), substantial toxicity, sometimes life-threatening, has lessened its widespread appeal (Siegel and Puri, 1991). While many groups are evolving to a new era of adoptive immunotherapy using gene-modified tumour or lymphoid cells in combination with rIL-2 (Rosenberg, 1992; Crowley and Seigler, 1993), it is unfortunate that at the end of its first decade we are not much wiser about the mechanism of rIL-2 toxicity or its potential modulation.

The pathophysiology of rIL-2-induced toxicity is associated with increased membrane permeability that leads to fluid and protein losses into visceral and soft tissues (Rosenthein *et al*., 1986), a so-called vascular leak syndrome. High-dose rIL-2 administration is associated with decreased systemic vascular resistance, an increase in cardiac index, neutrophil leucocytosis, raised serum adrenocorticotrophic hormone and elevated serum levels of tumour necrosis factor alpha (TNF-α) interleukin 1 (IL-1), and gamma-interferon (IFN-γ) (Siegel and Puri, 1991). The metabolic, physiological, immunological and endocrinological changes induced by rIL-2 closely simulate the septic state, particularly Gram-negative sepsis with endotoxaemia (Hack *et al*., 1991). The gastrointestinal tract has been identified as an occult source of sepsis in many critically ill patients (Goris *et al*., 1985; Deitch, 1992). Bacterial translocation, the passage of bacteria from an intact gastrointestinal tract to normally sterile tissues such as the mesenteric lymph nodes and liver, is central to this theory of gut-origin sepsis. The effects of rIL-2 on this phenomenon have not been described: the focus of this study was to address if some of the toxicity of high-dose rIL-2 therapy could relate to translocation of Gram-negative bacteria.

Correspondence: J Reynolds, Department of Surgery, 8th Floor, Clinical Sciences Building, St. James University Hospital, Leeds LS9 7TF, UK
Received 6 January 1995; revised 10 April 1995; accepted 13 April 1995

### Materials and methods

### *Experimental groups and protocol*

Fifty-one male Sprague–Dawley rats weighing 200–250 g were studied. Endotoxin-free human recombinant interleukin 2 (Eurocetus, The Netherlands) was used. There were five treatment groups: group A ($n = 15$), rIL-2, $10^5$ IU twice daily (b.d.) by intraperitoneal (i.p.) injection in 0.4 $cm^3$ of saline; group B ($n = 8$), rIL-2, $10^4$ IU b.d.; group C ($n = 8$), rIL-2, $10^3$ IU b.d.; group D ($n = 8$), rIL-2, $10^2$ IU b.d.; and group E ($n = 12$), saline alone, 0.4 $cm^3$ i.p. b.d.

All animals received a total of ten injections over 5 days. They were then sacrificed by cervical dislocation. The ileum was excised for routine light microscopy. The mesenteric lymph node (MLN) complex was carefully excised for bacteriological studies.

### *Microbiological assays*

The MLN complex was homogenised in Ringer's solution, and plated at multiple dilutions on McConkey agar and on blood agar. Plates were cultured for 48 h under aerobic (McConkey and blood agar) and anaerobic (blood agar) conditions. MLN colonisation is expressed as the number of colony-forming units (c.f.u.) per ml of homogenate. Colony-forming units were counted to an arbitrary maximum of $10^5$.

### *Statistical analysis*

Statistical significance was computed using the Mann–Whitney *U*-test.

### Results

There was no mortality in any group. There was no signigicant difference in body weight changes between groups *Escherichia coli* was the sole organism isolated in all but two colonised nodes, these containing in addition *Proteus mirab-*

*ilis* and *Streptococcus faecalis*. The incidence of *E. coli* colonisation of mesenteric nodes and the mean c.f.u. per ml of cultured nodes is shown in Table I. No saline-treated rat had evidence of bacterial colonisation of the MLN complex. For rIL-2-treated groups, there was a dose-dependent increase in colonised nodes, with all rats in the group receiving the highest dose of rIL-2 having *E. coli* colonisation of the MLN complex. This was significantly ($P<0.001$) greater than in all other groups. Furthermore, intense colonisation of nodes was observed only in this group with consistent yields of $>10^5$ c.f.u. per ml of homogenate. Nodal yields of *E. coli* in other groups varied from 5 to $10^3$ c.f.u. $ml^{-1}$.

Histopathological changes in villus architecture were observed only in rats receiving $10^5$ IU of rIL-2. A marked increase in submucosal vessels, most probably lymphatics, was observed in all rats in this group. Four rats in this group had in addition evidence of mucosal disruption with loss of epithelial cells (Figure 1).

### Discussion

The theory that translocation of bacteria and endotoxin from the gastrointestinal tract may initiate or exacerbate septic states is increasingly accepted (Van Leeuwen *et al*., 1994). In this so-called *gut hypothesis of sepsis and multiple organ failure*, translocated enteric bacteria and endotoxin induce cytokine secretion from tissue macrophages, stimulate neutrophil responses and promote a proinflammatory endothelial cell phenotype; organ injury may be mediated by complement and coagulation system activation as well as the products of activated phagocytes and neutrophils, including reactive oxygen intermediates, cytokines and proteases (Deitch, 1992). This theory may explain why no septic focus is identified in approximately 30% of bacteraemic patients dying of sepsis (Goris *et al*., 1985). Furthermore, gut-derived bacteria or endotoxin may fuel this immunoinflammatory septic state in the absence of microbiological evidence of infection.

**Table 1** Incidence of *E. coli* colonisation of the MLN complex and median c.f.u. $ml^{-1}$ per colonised complex in each group

| Group | *rIL-2 (IU)* | *Incidence*[a] *no. total (%)* | *Median (range) c.f.u.* $ml^{-1a}$ |
|---|---|---|---|
| A | $10^5$ | 15 15 (100) | $>10^5$ ($>10^5$ all rats) |
| B | $10^4$ | 5 8 ( 62) | 600 (5–$10^3$) |
| C | $10^3$ | 3 8 ( 37) | 20 (10–60) |
| D | $10^2$ | 1 8 ( 12) | 15 (15) |
| E | – | 0 12 ( 0) | – |

[a]Group A is significantly different ($P<0.001$) from all other treatment groups with respect to incidence of MLN colonisation and median c.f.u. $ml^{-1}$; $P<0.0001$ group A *vs* E for both these parameters.

**Figure 1** High-power ( × 25) photomicrograph of villi from rat receiving 105 IU of rIL-2 b.d. showing dilated lymphatics and mucosal disruption with loss of epithelial cells.

Experimental studies have shown that the mesenteric lymph node is the most reliable site to culture for the purposes of monitoring bacterial translocation. Our experimental design focused on this parameter and clearly shows a dose-dependent increase in bacterial translocation with rIL-2 administration. All colonised lymph nodes contained *E. coli* and hence endotoxin. Although rIL-2 at concentrations of $10^2$ IU–$10^4$ IU b.d. produced translocation, the bacterial yield per ml of homogenised nodes was markedly less than in the group of 15 rats who received $10^5$ IU b.d., all of which grew more than 100 000 *E coli* c.f.u. $ml^{-1}$.

The mechanism of rIL-2-induced translocation is unknown. Usually one or more conditions are necessary for bacterial translocation to occur (Wilmore *et al*., 1988): (1) physical disruption of the mucosal barrier; (2) altered ecological balance of the gastrointestinal flora, most usually with Gram-negative overgrowth; and (3) impaired immune defences. Mucosal disruption was observed using light microscopy in 4 of 15 rats receiving high-dose rIL-2, and all rats in this group had dilated submucosal vessels, probably lymphatics. There was no evidence of substantially increased tissue oedema, suggesting that increased microvascular permeability in the gut is unlikely to be a significant component. We are currently evaluating flora and assessing immune function in these groups. We do not know if high-dose rIL-2 promotes bacterial translocation via a direct action on the gastrointestinal tract or secondary to gut microcirculatory or inflammatory changes consequent on the generation of substantial local proinflammatory cytokines and other mediators. For neutrophils to mediate injury, adhesion to endothelial cells is a prerequisite. In man the intercellular adhesion molecule 1 (ICAM-1), the ligand for CD11a, is induced *de novo* in skin biopsies of patients treated with high-dose rIL-2 (Cotran *et al*., 1987). High-dose rIL-2 induces TNF-$\alpha$ and IFN-$\gamma$, which contribute to the toxic effects of rIL-2 (Economou *et al*., 1991). Furthermore, other experimental studies have correlated tissue injury induced by rIL-2 with raised levels of the proinflammatory mediators thromboxane $B_2$ (Welbourn *et al*., 1991) and leukotriene $B_4$ (Klausner *et al*., 1990). Since rIL-2 does not produce increased vascular permeability in nude mice, it is very likely that indirect or secondary events with gastrointestinal or systemic immune cells are of greatest importance. Irrespective of the mechanism of translocation, once bacterial and endotoxin translocation is initiated, a self-perpetuating cycle of translocation may be sustained, because *E. coli* endotoxin is itself a potent promoter of translocation (Deitch, 1992).

Do these data have clinical relevance? Although *E. coli* bacteraemia is well described, the most common organisms causing nosocomial bacteraemia in patients on high-dose rIL-2 are *Staphylococcus aureus* and *Staphylococcus epidermis* (Snydman *et al*., 1990). Gastrointestinal side-effects are, however, extremely common in patients receiving high-dose rIL-2, occurring in 85% of patients, and intestinal necrosis and perforation have been described (Rahman *et al*., 1991). We suggest that translocation may fuel toxicity in man without producing microbiological evidence of infection. Most patients with rIL-2 toxicity have features of sepsis, despite negative systemic cultures; *E. coli* and endotoxin may activate the host neutrophils and phagocytes to produce the immunoinflammatory mediators responsible for the sepsis syndrome. The hepatic mononuclear phagocyte, the Kuppfer cell, may be a key regulatory player, contributing to the manifestations of sepsis through cytokine elaboration and limiting the passage of *E. coli* and endotoxin from the portal to the systemic circulation.

In conclusion, we have observed for the first time that high-dose rIL-2 produces bacterial translocation in rats. These observations may enable us to evaluate strategies directed at decreasing toxicity of high-dose rIL-2, either by decreasing bacterial and endotoxin translocation or limiting the consequences of endotoxaemia. Selective gut decontamination, early enteral nutrition, glutamine supplementation, polymyxin B, anti-endotoxin and anti-TNF antibodies have theoretical rationale (Deitch, 1992).

### References

COTRAN RS, POBER JS AND GIMBRONE MA. (1987). Endothelial activation during interleukin-2 immunotherapy. A possible mechanism for the vascular leak syndrome. *J. Immunol.*, **139**, 1883–1888.

CROWLEY NJ AND SEIGLER HF. (1993). Possibilities of immunotherapy and gene therapy for malignant melanoma. *Semin. Surg. Oncol.*, **9**, 273–278.

DEITCH EA. (1992). Multiple organ failure. Pathophysiology and potential future therapy. *Ann. Surg.*, **216**, 117–134.

ECONOMOU JS, HOBAN M, LEE JD, ESSNER R, SWISHER S, McBRIDE W, HOON DB AND MORTON DL. (1991). Production of tumour necrosis factor alpha and interferon gamma in interleukin-2-treated patients: correlation with clinical toxicity. *Cancer Immunol. Immunother.*, **34**, 49–52.

GORIS RJ, BEOKHORST PA AND NUTYNICK KS. (1985). Multiple organ failure: generalised autodestructive inflammation. *Arch. Surg.*, **120**, 1109–1115.

HACK CE, WAGSTAFF J, STRACK VAN SCHIJNDEL RJ, EERENBERG AJ, PINEDO HM, THIJS LG AND NUIJENS JH. (1991). Studies on the contact system of coagulation therapy with high doses of recombinant IL-2: implications for septic shock. *Thromb. Haemost.*, **65**, 497–503.

KLAUSNER JM, GOLDMAN G, SKORNICK V, VALERI R, INBAR M, SHEPRO D AND HECHTMAN HB. (1990). Interleukin-2-induced lung permeability is mediated by leukotriene $B_4$. *Cancer*, **66**, 2357–2364.

RAHMAN R, BERNSTEIN Z, VAICKUS L, PENETRANTE R, ARBUCK S, KOPEC I, VESPER D, DOUGLASS HO AND FOON KA. (1991). Unusual gastrointestinal complications of interleukin-2 therapy. *J. Immunother.*, **10**, 221–225.

ROSENBERG SA. (1992). The immunotherapy and gene therapy of cancer. *J. Clin. Oncol.*, **10**, 180–189.

ROSENTHEIN M, ETTINGHAUSEN SE AND ROSENBERG SA. (1986). Extravasation of intravascular fluid mediated by the systemic administration of recombinant interleukin-2. *J. Immunol.*, **137**, 1735–1742.

SIEGEL JP AND PURI RK. (1991). Interleukin-2 toxicity. *J. Clin. Oncol.*, **9**, 694–704.

SNYDMAN DR, SULLIVAN B, GILL M, GOULD JA, PARKINSON DR AND ATKINS MB. (1990). Nosocomial sepsis associated with interleukin-2. *Ann. Intern. Med.*, **112**, 102–107.

TANIGUCHI T, MATSUI H AND FUJITA T. (1983). Structure and expression of a cloned cDNA for human interleukin-2. *Nature*, **302**, 305–307.

VAN LEEUWEN PAM, BOERMEESTER MA, HOUDIJK APG, FERWERDA C, CUESTA MA, MEYER S AND WESDORP RIC. (1994). Clinical significance of translocation. *Gut*, **(suppl. 1)**, S28–S34.

WELBOURN R, GOLDMAN G, KOBZIK L, PATERSON I, SHEPRO D AND HECHTMAN HB. (1990). Interleukin-2 induces early multisystem organ oedema mediated by neutrophils. (1991). *Ann. Surg.*, **214**, 181–186.

WILMORE DW, SMYTH RJ AND O'DWYER ST. (1988). The gut: a central organ after surgical stress. *Surgery*, **104**, 917–923.